# Brucellosis Mimicking COVID-19: A Point of View on Differential Diagnosis in Patients With Fever, Dry Cough, Arthralgia, and Hepatosplenomegaly

**DOI:** 10.7759/cureus.15848

**Published:** 2021-06-23

**Authors:** Gultekin Ozan Kucuk, Selim Gorgun

**Affiliations:** 1 Department of General Surgery, University of Health Sciences, Samsun Education and Research Hospital, Samsun, TUR; 2 Department of Microbiology, University of Health Sciences, Samsun Education and Research Hospital, Samsun, TUR

**Keywords:** brucella melitensis, brucellosis, hepatomegaly, coronavirus disease 2019 (covid-19), endemic infections

## Abstract

The novel Coronavirus disease 2019 (COVID-19), caused by severe acute respiratory syndrome Coronavirus-2, emerged in China in late 2019, and a variety of clinical symptoms and signs were reported following patients’ clinical presentation. By contrast, human Brucellosis is a worldwide zoonosis that may present with general symptoms including fever, dry cough, malaise, and arthralgia, making it indistinguishable from other causes of respiratory infection. Here, an 18-year-old man who was hospitalized with a suspected COVID-19 infection, but finally confirmed as having Brucellosis with positive blood culture for Brucella melitensis is presented. This case is a reminder for healthcare workers to consider the diagnosis of Brucellosis in patients exhibiting febrile syndromes in endemic regions during the COVID-19 pandemic.

## Introduction

The pandemic caused by severe acute respiratory syndrome Coronavirus-2 (SARS-CoV-2) remains a global concern well into mid-2021. SARS- CoV-2 infection may involve multiple tracts of the body, including respiratory, gastrointestinal, musculoskeletal, and neurologic systems [1]. Due to this variety of numerous system involvement, controversies remain concerning the clinical presentation of Coronavirus disease 2019 (COVID-19). The co-existence of respiratory symptoms with other systemic manifestations due to some endemic and regional pathogens may lead to a misdiagnosis and delay in the proper treatment of the causative agent. In this article, a patient who was hospitalized a suspected case of COVID-19, due to symptoms of fever, dry cough, and lower extremity pain, but who was finally diagnosed with Brucellosis following a positive blood culture for Brucella melitensis is discussed.

## Case presentation

An 18-year-old male patient was admitted to the emergency department following a two-day history of fever, cough, shortness of breath, and malaise. On admission, there was no confirmed case of COVID-19 in his family. The patient had a history of operation due to urolithiasis which was performed 10 years ago. In the emergency clinic, his workup included a computerized chest tomography (CT) scan with a result of no finding suggesting COVID-19 infection. On admission, his body temperature was 37.5 °C. Laboratory results from the emergency department showed a normal white blood cell count (4.5 x 10^9^/L), a hemoglobin level of 12.4 g/dl, a low platelets count (89,000 per mm3), elevated transaminases (Aspartate aminotransferase level of 133 U/L, alanine aminotransferase level of 100 U/L), and a high c-reactive protein concentration (66.54 mg/L). After an initial evaluation, he was suspected of having a COVID-19 and was hospitalized on account of his high body temperature, dry cough, and dyspnea. In the clinic, a reverse transcription real-time fluorescence polymerase chain reaction (RT-PCR) with nasopharyngeal swab culture was taken, and the drugs hydroxychloroquine (400 mg/2 times on day 1 and then 200 mg/2 times per day for 4 days) and azithromycin (500 mg/2 times on Day 1 and then 250 mg/2 times per day for 4 days) were administered as empirical treatments.

On the second day of hospitalization, his fever was measured at 39.6°C, and a blood culture was sent to the microbiology laboratory. The first swab culture RT-PCR result for COVID-19 infection was found negative, and a second swab culture was taken. The second swab culture for COVID -19 reported a negative result, as well.

**Figure 1:**
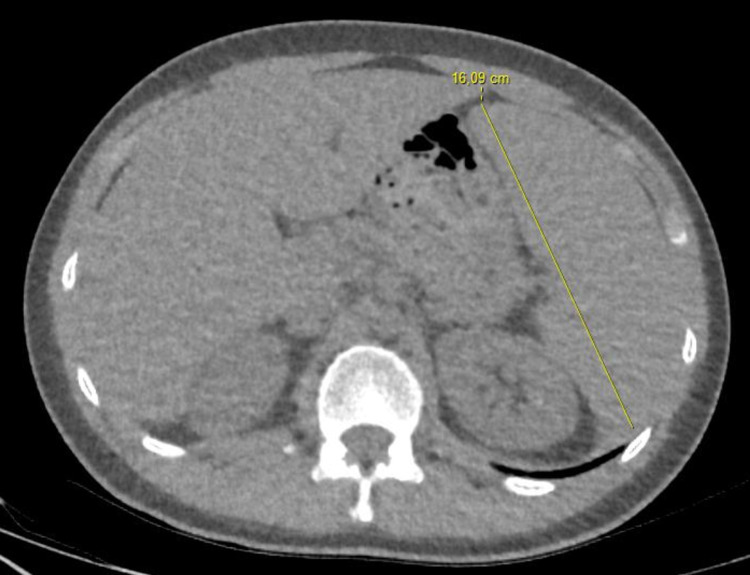
CT image of patient with hepatosplenomegaly.

On the fourth day of hospitalization, the patient complained of pain in his left knee. Paracetamol 3x500 mg was added to his treatment. After five days of hospitalization, he was discharged with advice to quarantine at home for l4 days. At the time of discharge, his temperature and other vitals were normal. His respiratory symptoms and pain in the lower extremities had improved. Two days later, after his discharge, the blood culture reported *Brucella melitensis.* Further, the radiologic images, including the thorax CT, was re-assessed and hepatosplenomegaly was detected in lower sections of the thorax CT (Figure 1). Brucellosis was diagnosed based on clinical, laboratory, and radiological findings and the patient’s history. The patient was re-called for further clinical evaluation and treatment.

## Discussion

Brucellosis is an important health problem for people living in Asia, the Mediterranean Basin, Africa, South America, Eastern Europe, and the Middle East [2]. People may become infected with the bacteria after close contact with an animal or the consumption of unpasteurized dairy products infected by Brucella [3].

The patient’s medical history is crucial in the clinical diagnosis of the disease. In this case, one patient with chief complaints of fever, dry cough, and shortness of breath in an emergency was suspected of having COVID-19 and hospitalized. During the COVID-19 pandemic, taking a detailed medical history was not always possible, due to the risk of transmitting the virus. In our case, when a detailed further history of the patient was taken, after the positive blood culture for Brucella, it was found that the patient's family owns a sheep farm and his father had a contact history with an aborted sheep fetus nearly 40 days prior. Although pulmonary Brucellosis is rare, patients with brucellosis may exhibit respiratory symptoms (mainly dry cough) without no radiologic documentation [4]. Additionally, it may result in pneumonia, pleural effusion, abscess of lungs, granulomas, solitary nodules, pleural effusion, interstitial pattern, and empyema in endemic countries [5]. However, there was no finding indicating pulmonary brucellosis in our patient’s thoracic CT. 

In patients presenting to the emergency department with fever, it is important to comment not only on the lung pathology, but also on the liver and spleen in lower thoracic sectional images, as these may give some clues about the etiology of the fever. Supporting these CT findings with increased transaminases may help to review differential diagnoses in patients with respiratory findings and fever.

## Conclusions

Brucellosis, a zoonotic infection, is an important public health problem in many developing countries. It is important to collect information from the patient regarding possible Brucella exposure, especially in countries that are accepted as endemic for Brucellosis. This etiology should be considered during the differential diagnosis of patients presenting with fever, shortness of breath, and dry cough in endemic regions during the COVID-19 pandemic.
